# Metabolic Syndrome Remodels Electrical Activity of the Sinoatrial Node and Produces Arrhythmias in Rats

**DOI:** 10.1371/journal.pone.0076534

**Published:** 2013-11-08

**Authors:** Alondra Albarado-Ibañez, José Everardo Avelino-Cruz, Myrian Velasco, Julián Torres-Jácome, Marcia Hiriart

**Affiliations:** 1 Departamento de Neurodesarrollo y Fisiología, Instituto de Fisiología Celular, Universidad Nacional Autónoma de México. México D.F., México; 2 Laboratorio de Cardiología Molecular, Instituto de Fisiología, Benemérita Universidad Autónoma de Puebla, Puebla, Puebla, México; 3 Laboratorio de Fisiopatología Cardiovascular, Instituto de Fisiología, Benemérita Universidad Autónoma de Puebla, Puebla, Puebla, México; University of North Carolina at Chapel Hill, United States of America

## Abstract

In the last ten years, the incidences of metabolic syndrome and supraventricular arrhythmias have greatly increased. The metabolic syndrome is a cluster of alterations, which include obesity, hypertension, hypertriglyceridemia, glucose intolerance and insulin resistance, that increase the risk of developing, among others, atrial and nodal arrhythmias.

The aim of this study is to demonstrate that metabolic syndrome induces electrical remodeling of the sinus node and produces arrhythmias. We induced metabolic syndrome in 2-month-old male Wistar rats by administering 20% sucrose in the drinking water. Eight weeks later, the rats were anesthetized and the electrocardiogram was recorded, revealing the presence of arrhythmias only in treated rats. Using conventional microelectrode and voltage clamp techniques, we analyzed the electrical activity of the sinoatrial node.

We observed that in the sinoatrial node of “metabolic syndrome rats”, compared to controls, the spontaneous firing of all cells decreased, while the slope of the diastolic depolarization increased only in latent pacemaker cells. Accordingly, the pacemaker currents I_f_ and I_st_ increased. Furthermore, histological analysis showed a large amount of fat surrounding nodal cardiomyocytes and a rise in the sympathetic innervation. Finally, Poincaré plot denoted irregularity in the R-R and P-P ECG intervals, in agreement with the variability of nodal firing potential recorded in metabolic syndrome rats.

We conclude that metabolic syndrome produces a dysfunction SA node by disrupting normal architecture and the electrical activity, which could explain the onset of arrhythmias in rats.

## Introduction

Sedentary lifestyle and fast food have positioned the metabolic syndrome (MeS) as the epidemic of this century [1], [2]. MeS, increase the risk factor for developing diabetes mellitus type 2 (DM2), and cardiovascular diseases [3], [4]. MeS also increases the probability of suffering supraventricular arrhythmias, like in the Sick Sinus Syndrome (SSS) and the abnormal P-wave. Moreover, the risk of developing atrial fibrillation increases in patients with obesity and MeS [5], [6], [7]. Atrial fibrillation is the most common tachyarrhythmia associated with an increased morbidity and mortality [7],[8],[9].

The precise mechanisms through which MeS causes atrial fibrillation are not completely understood, but the syndrome has been associated with electrical remodeling of the atrium and sinoatrial (SA) node [8], [10]; specifically, an increase in action potential duration and changes in the refractory period, as well as changes in the passive electrical properties in both tissues [11], [12], [13].

Indeed, it has been demonstrated that old obese rats undergo electrical remodeling of pacemaker activity [14]. Furthermore, Lin et al proposed that excessive deposits of fat on the heart could cause electrical remodeling of the atrium, since adipocytes on the epicardial surface interact with cardiomyocytes. This generates ectopic excitation foci and increases oxidative stress because adipose tissue releases adipokines and pro-inflammatory cytokines [15], [16].

In the same way, mortal arrhythmias are associated with a reduced sympathetic activity or with a reduced vagal tone [7], [17]. In fact, the quantification of sympathetic and vagal activity, taken together with the study of heart rate variability has been proposed as a marker for supraventricular arrhythmias [18], [19]. Actually, heart rate variability is considered one of the best markers of AF by cardiologists [20].

Currently, there are no specific studies correlating MeS and cardiac alterations. Most studies have correlated the individual conditions of MeS and the risk to develop cardiovascular disease [1], [2]; however, specific cardiac alterations induced by MeS have not been reported. In this work we investigated if MeS produces electrical remodeling of the SA node and if these changes predispose nodal tissue to suffer arrhythmias that could eventually spread to the atrium.

## Materials and Methods

### Animals

All methods used in this study were approved by the Animal Care Committee of the Instituto de Fisiología Celular, Universidad Nacional Autónoma de México. Animal care was to the “International Guiding Principles for Biomedical Research Involving Animals”, Council for International Organizations of Medical Sciences, 2010. Wistar rats were obtained from the local animal facility, maintained with a 12∶12 h light-dark cycle (0600-1800), and allowed free access to standard laboratory rat diet and tap water.

### Metabolic syndrome model

We used 2-months old male Wistar rats weighing between 250 and 280 g. Metabolic syndrome was induced by including 20% sucrose in the drinking water for 8 weeks [21]. After treatment, we took a blood sample to measure glucose, triglycerides, cholesterol, fatty acids and HDL-c. Additionally, central obesity, body weight and epididymal fat were analyzed.

### Electrocardiography

An electrocardiogram (ECG) was performed on rats (8 control animals and 7 with MeS) anesthetized with sodium pentobarbital (63 mg.Kg^−1^, IP). Bipolar ECGs were recorded using subcutaneous needle electrodes and following ECG lead I configuration. ECG signal was 700X amplified and acquired at 1 KHz for 5 minutes. Data was stored in a personal computer and analyzed off-line using ClampFit (Molecular Devices). All rats were continuously monitored to guarantee right ventilation and temperature.

### Sinoatrial node dissection

SA node was prepared as previously reported [22]. Rats were heparinized (1000 U/kg IP) and anesthetized with sodium pentobarbital (63 mg.kg^−1^, IP). The heart was immediately excised, hung up and perfused in a Langendorff system at 36.5°C.

For the isolated atrial preparation the ventricles were removed and the atria were stretched and pinned to the bottom of a sylgard-coated chamber (Figure S1). Once the SA node was cleaned of surrounding tissues, it was fixed with tungsten pins to a sylgard-based chamber with the endocardial face up. Tissue was left resting for three hours and then spontaneous action potentials (AP) were recorded.

### Action potential recordings in sinoatrial node preparation

The AP of SA nodes were recorded using sharp microelectrodes of borosilicate (WPI Inc.) filled with 3M KCl and resistance of 25–35 MΩ. The sensed signal was amplified with a WPI Duo 776 electrometer, digitalized (SCB-68 Quick Reference label, National Instruments), and captured at 10 KHz on a personal computer where it was analyzed using ClampFit (Molecular Devices) and Origin 7.0 (Southampton) [23].

To record spontaneous electrical activity we first localized the nodal artery on the SA node [22], and the electrical activity was recorded for 60 seconds. Using the same preparation we explored the whole sinoatrial node, as explained in Figure S1, every AP was associated with a recording position.

### Isolation of sinoatrial node cells

Sinoatrial nodal cells were isolated as reported by Aréchiga-Figueroa, et al., 2010. Briefly, rats were anesthetized and heparinized, and the heart was excised and mounted via the aorta to a Langendorff perfusion system. After Tyrode solution perfusion, the heart was digested with enzymatic method for 12 minutes in calcium-free solution. Then, the SA node was dissected out from the heart, cut into small pieces and incubated in 0.5 mg/mL collagenase and 0.2 mg/mL elastase at 36°C, for at least 5 minutes. Finally, we proceeded with mechanical dissociation in Kraft-Brüeh (KB) solution.

### Voltage clamp experiments

We used the patch clamp technique in whole cell configuration to record pacemaker currents, the patch pipettes had a resistance between 2 and 4 MOhm. The signal was captured at 10 KHz, amplified (Axopatch 1C, Axon Instrument Inc.), digitalized (Digidata TL1 interface, Axon Instrument Inc.), and stored on a personal computer. Recordings were analyzed off-line using pClamp 9.2 (Molecular Devices) and Origin 8.0 (OriginLab corporation).

The cardiomyocytes were placed in a perfusion chamber mounted to an inverted microscope (Axioscope, Nikon). Only beating, spindle-shaped cells were selected for the study in order to avoid atrial cardiomyocytes. The capacitances of cells were ranged from 20 to 65 pF; these values are similar to those reported for nodal cells [24].

### Protocol currents

The funny current (I_f_) was evoked by square voltage pulses with a holding potential of −40 mV and going from −20 mV to −120 mV in 10 mV increments. The initial step lasted 2.5 seconds and successive pulses were decreased in 200 ms, time between pulses was 20 seconds [23], [24].

The potassium current (I_k1_) was elicited from a holding potential of −60 mV by square voltage pulses lasting 1 second and going from −60 mV to −120 mV in 10 mV increments, every 15 seconds.

The sustained current (I_st_) is an unspecific cationic was fixed the membrane potential at −80 mV then applied hyperpolarizing potentials from −80 mV to −120 mV for 500 ms every 10 seconds.

### Solutions

The *Tyrode solution* contained (mM) 118 NaCl, 5.4 KCl, 1.05 MgCl_2_, 0.42 NaH_2_PO_4_, 1.8 CaCl_2_, 24 NaHCO_3_ and 11 glucose. The solution was equilibrated with 95% O_2_ and 5% CO_2_ with pH of 7.4 [23].

The *Tyrode calcium-free* solution was prepared omitting CaCl_2_ in the Tyrode solution.

The *Kraft-Brüeh (KB)* solution contained (mM): 80 K-glutamate, 40 KCl, 10 KH_2_PO_4_, 20 taurine, 0.2 EGTA, 10 HEPES, 0.5 creatine, 5 MgSO_4_ and 10 succinic acid; pH was adjusted to 7.4 with KOH [23]. The solution was saturated with 100% O_2_.

The *external patch solution* to record I_f_, I_k1_ and I_st_ currents had the following composition (mM): 136 NaCl_2_, 4 KCl, 2 MgCl_2_, 11 glucose, 10 HEPES, and 1.8 CaCl_2_; pH was adjusted to 7.4 with NaOH.

To record I_f_ or I_k1_ currents the solutions were supplemented with 0.5 mM BaCl_2_ or 1 mM CsCl, respectively [24]. In the case of I_st_ recordings, the solution was supplemented, either with 0.5 mM BaCl_2_ or 1 mM CsCl.

Patch pipettes were filled with *internal solution* with the following composition (mM): 80 potassium aspartate, 10 KH_2_PO_4_, 1 MgSO_4_.7 H_2_O, 40 KCl, 10 HEPES, 10 EGTA, 3 Na_2_ATP and 0.2 NaGTP; pH adjusted to 7.3 with KOH.

### Immunofluorescent staining

Indirect immunostaining was analyzed using confocal microscopy (Confocal Olympus FV1000, Olympus America Inc.). SA nodes were isolated as mentioned above, embedded in Tissue-Tek (Sakura), frozen, and cut coronally into 5 µm-thick slices beginning from endocardium. The antibodies used were anti-tyrosine hydroxylase (1∶250 rabbit polyclonal antibody; Millipore Corporation) and CY-5 (1∶200, rabbit polyclonal antibody; Jackson ImmunoResearch laboratory Inc.).

### Lipid staining

The oil red-O stain was used to detect hydrophobic lipids (cholesteryl ester and triglycerides). The frozen sections were fixed with formalin for 3 minutes, then washed three times with PBS and placed in absolute propylene glycol for 4 minutes. Staining was by immersion in a warm red-oil solution for 10 minutes, and then sections were washed in 85% propylene-glycol for 3 minutes. After washing, a counterstaining was performed with Gill's hematoxylin. Finally, slices were mounted in glycerin jelly.

### Rate variability

To quantify the variation in heart rate and the firing rate nodal we used a Poincaré plot constructed with R-R and P-P intervals of the electrocardiogram and the interpotential intervals of the spontaneous AP respectively. To construct the Poincaré plot was plotted the second interval I(n+1) as function of the first I(n). To quantify the variability, we calculated standard deviation of the distances between all points of the diagram and the line I(n+1) = I(n), this value is called SD1 or width of the plot. In the same way, the standard deviation of the distances between the points of the graph and the line I(n+1) = −I(n)+2I(n) is called SD2 or length. SD1/SD2 is the index of rate variability [18], [19], [20].

### Action potential analysis

A file corresponding to one minute of spontaneous activity of sinoatrial node was opened in pClamp 9.2 software (Axon Instruments) and it was calculated the following parameters amplitude, peak, rate of depolarization, upstroke velocity, action potential duration (APD), and frequency of firing, these parameters were used to classify the nodal action potential (See Figure S2).

### Patch clamp recording

The current at the end of each test pulse of the Protocol was measured for the current to voltage graphic; all values corresponding to each experimental condition were averaged and compared via a t-test. Final graphs were normalized by cell capacitance.

### Data analysis and statistics

All data is presented as the mean ± standard error unless otherwise specified. To compare the same variable of two types of AP we used the ANOVA-test. To assess differences between two metabolic conditions of the same variable we used the t-test. Values were considered statistically significant if p value was inferior to 0.05, denoted with an * or §.

## Results

### Metabolic parameters of the MeS model

**Figure 1:**
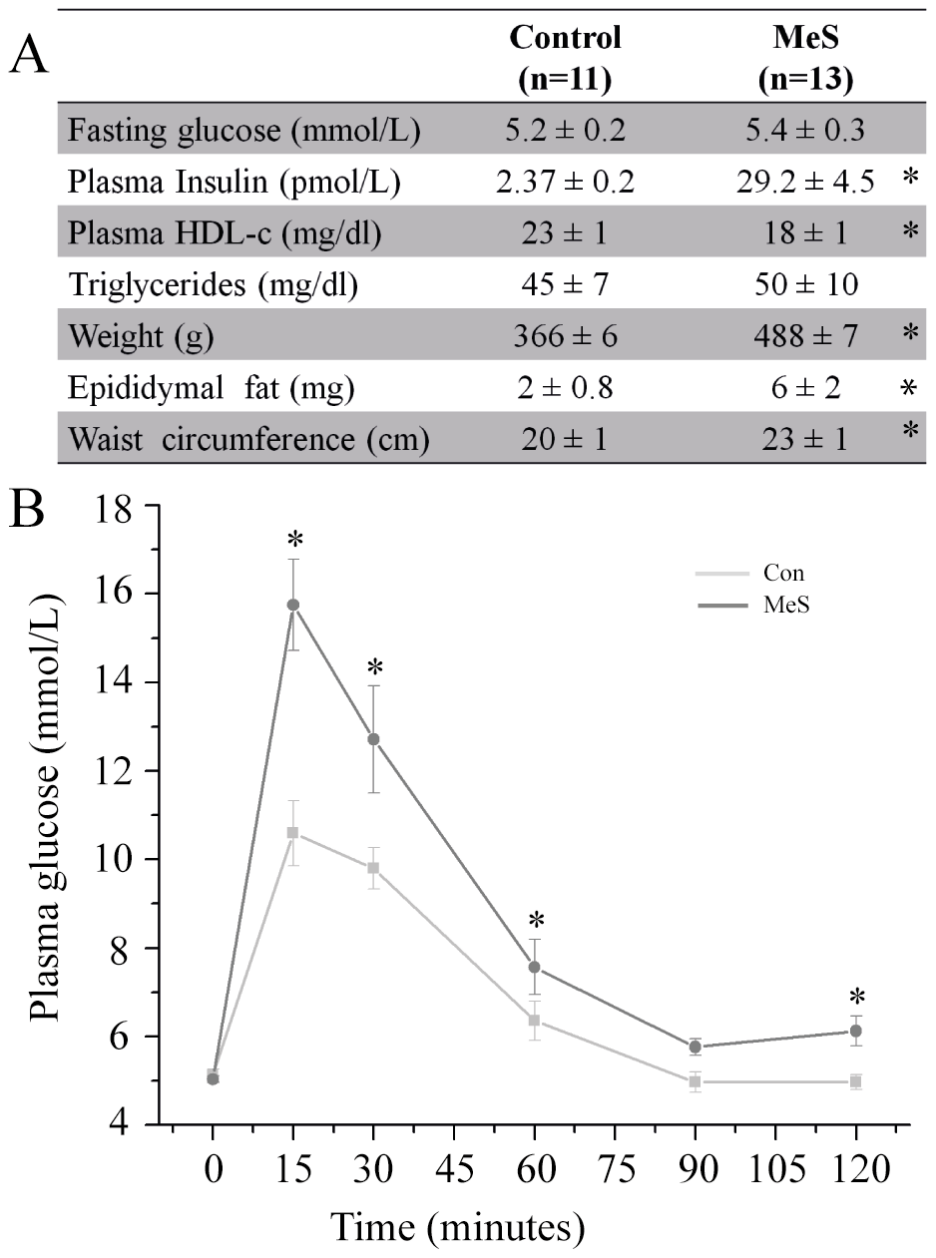
High carbohydrate diet produces MeS in Wistar rats. (**A**) Metabolic parameters associated with MeS. (**B**) Intraperitoneal glucose tolerance test in rats with MeS (control: n = 11; MeS: n = 13). * p≤0.05 vs. control.

The MeS group consisted of 13 rats, all of which presented at least three signs of the syndrome. Body weight and waist circumference increased by 23% and 14%, respectively, in controls and treated animals (p<0.05); epididymal fat, a central obesity marker in rats, increased by 3 times (from 2±0.8 g in controls to 6±2 g in the treated rats p<0.05) compared to their age-matched littermates in the control group. We also found a 10-fold increase in the insulin levels in plasma (Figure 1A). Additionally, an intraperitoneal glucose tolerance test revealed reduced glucose metabolism in MeS rats (Figure 1B).

### Electrocardiographic changes in MeS

**Figure 2:**
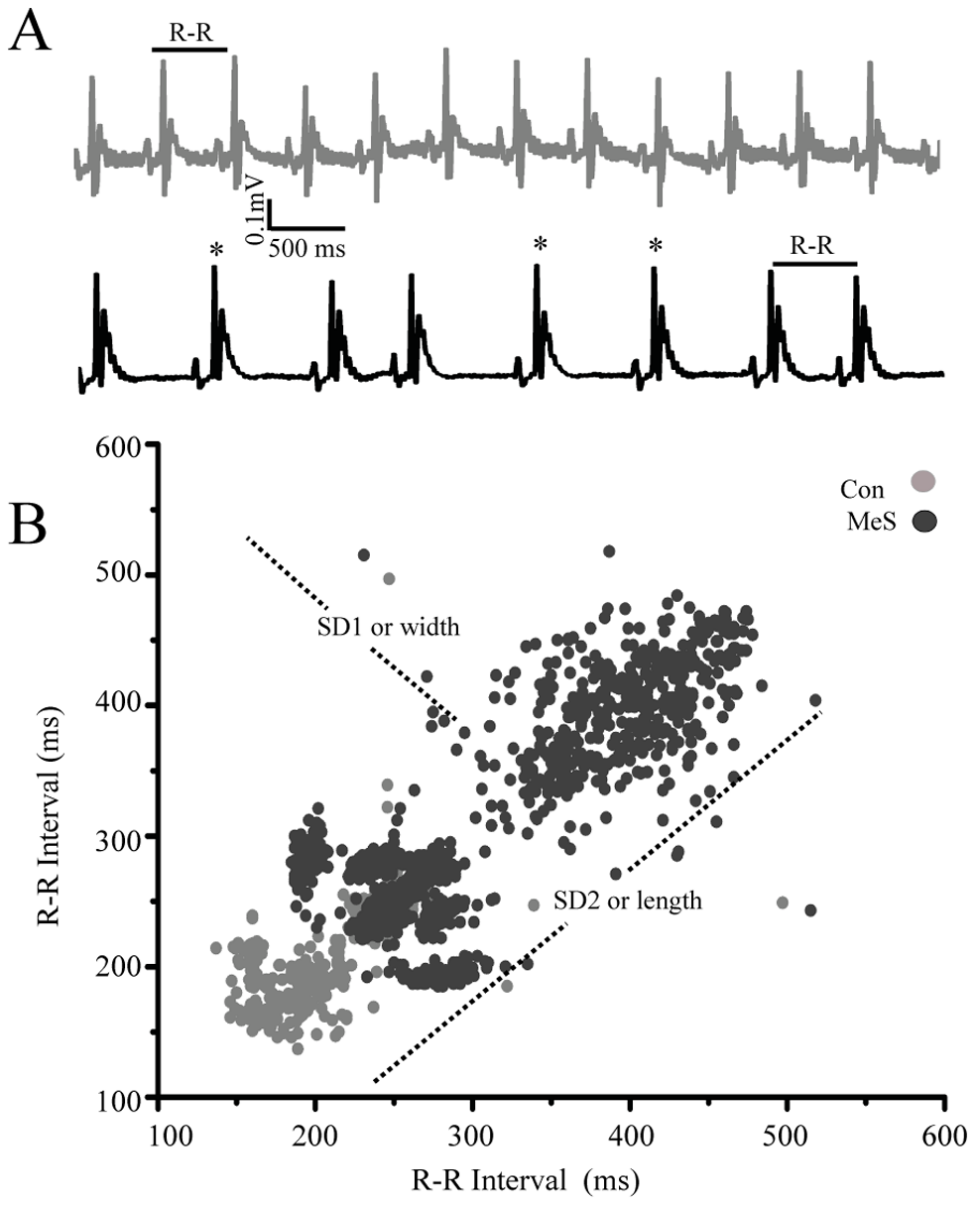
MeS increased risk of suffering arrhythmias. (**A**) Upper trace, representative electrocardiogram (ECG) of control rats. Lower trace; rats with MeS presented irregular sinusal rhythm (see asterisks). (**B**) Poincaré plot of ECG R-R interval evidenced an increase in the heart rate variability in MeS (n = 7; black) vs. control animals (n = 8; grey). The line dot represents the length and width of the area surrounding the points in Poincaré plot. (This plot was built with each interval R-R of ECG as a function of the previous R-R interval).

Electrocardiographic studies evidenced in the MeS model a lower heart rate (control = 4.2±0.01, n = 7 Hz vs. MeS = 3.7±0.7 Hz, n = 8) and an increase in beat variability compared to control rats. In addition, 43% of rats with MeS showed an increase in the R-R interval with an irregular pattern, consisting of a short interval followed by a long one, a pattern similar to the SA node firing recorded in vitro (Figure 2).

In the R-R Poincaré plot, the control standard deviations were, SD1 (5.5±0.1) and SD2 (26±0.5). In contrast in the MeS model were SD1 (15±0.4) and SD2 (69±1); 3 times increased compared to controls. However, SD1/SD2 quotients were not different, because SD1 and SD2 increased proportionally. The remaining 57% of the rats with MeS showed ventricular arrhythmias, registered as T wave inversion, bigeminy and short QT syndrome (data not shown). The mean QRS complex did not change, being 21±6 ms for controls and 22±6 ms for MeS. Interval QT was 55±10 ms and 42±2 ms respectively for control and MeS. The corrected QT was 3.6 ms for controls and 2.5 ms for MeS.

### Recording of spontaneous electrical activity of the sinoatrial node

**Figure 3:**
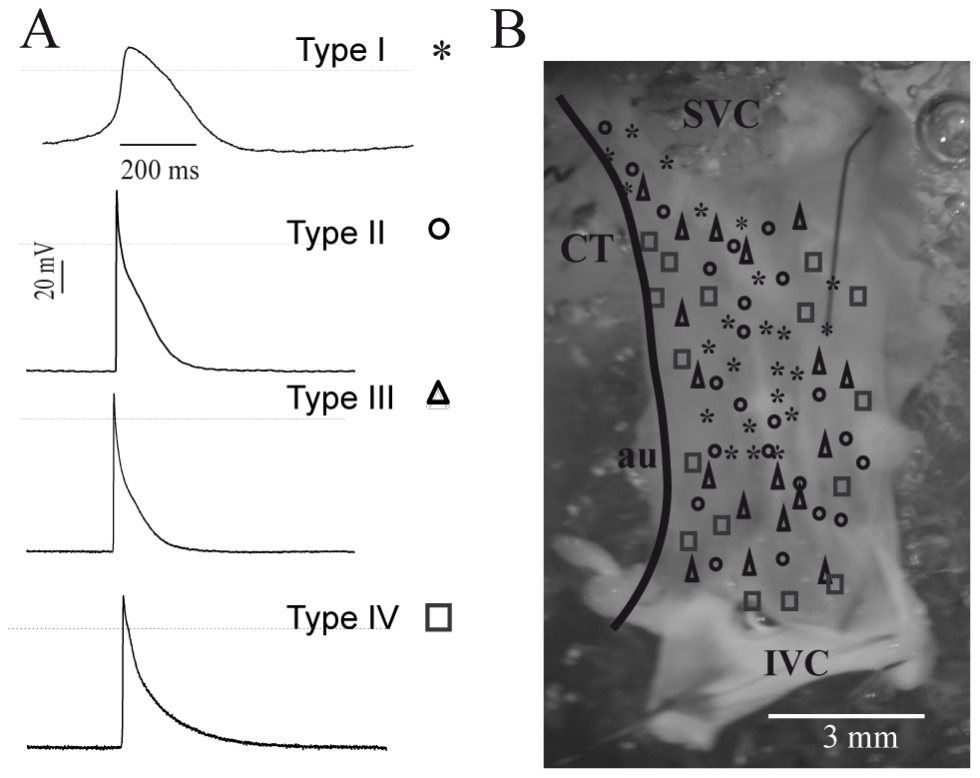
The distribution of activity electrical on sinoatrial node is heterogeneous. (**A**) Representative traces of AP type I, asterisk; latent type II, circle; type III, triangle and type IV, square. (**B**) Topological distribution on SA node AP's. SVC, superior vena cava; IVC, inferior vena cava; CT, *crista terminalis*; au, auricle.

In the spontaneous activity of the entire SA node, at least two different types of action potential (AP) were identified. One was a “true pacemaker” AP that has a phase 4, with slope of ∼10 V/s, an slow upstroke and repolarization, all classical parameters of true nodal cells [14], [24], [25]. The second type was characteristic of “latent pacemaker” cells, these AP have a shorter duration than true nodal AP or type I; the repolarization and upstroke phases were also faster. A detailed (Figure S2) analysis revealed that AP of latent cells can be divided in 3 subgroups (type II, III and IV). The type IV showed higher frequency, amplitude and depolarization rate than types III, II or even I. Moreover, type I had a longer duration than the rest of the subtypes (AP lasting: type I>II>III>IV), Figures 3A and S2.

### Spatial distribution of action potential in the sinoatrial node

The type I APs were located between the superior vena cava (SVC), close to the crista terminalis, to within nearly 6 mm of the mouth of the inferior vena cava (IVC). The distribution of AP shifts toward the center as it approaches the IVC. Type IV AP surrounded type I; type III latent cells almost overlap the distribution of type IV cells, but remained close to the center of the node. Finally, some type II APs were detected in the middle, overlapping type I cells, but most of the cells were dispersed along with type III and IV APs cells (Figure 3).

### MeS modify action potential morphology and increases firing variability of nodal tissue

**Figure 4:**
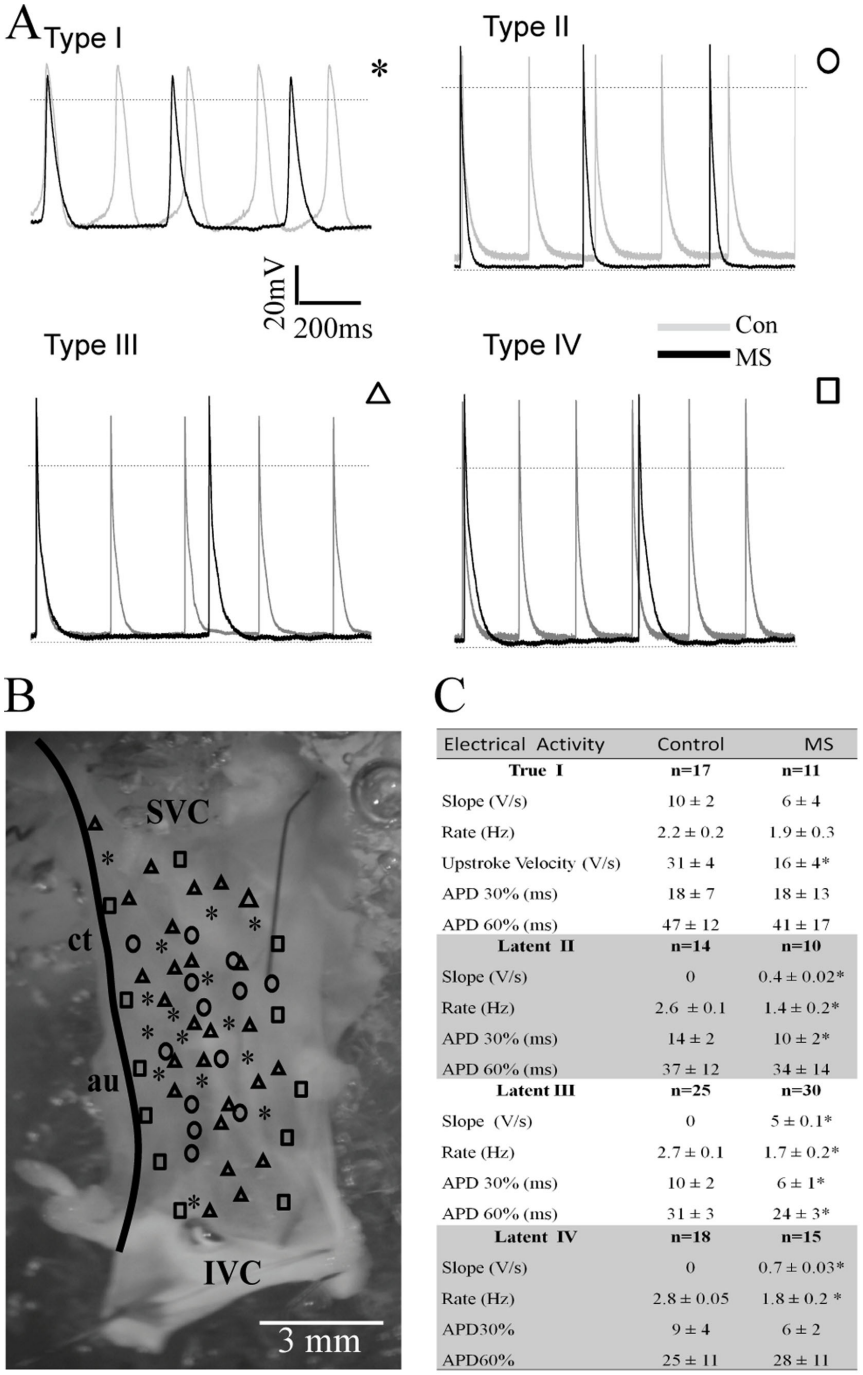
MeS remodels the electrical activity of sinoatrial node. (**A**) MeS decreases firing rate of true and latent pacemaker cells, (**B**) without any change in their location. (**C**). Note that true pacemaker cells in rats with MeS fire AP at higher frequencies than latent cells. § p≤0.05 vs. control.

The analysis of AP recorded in the nodal tissue from MeS rats did not reveal changes in the topological distribution reported above (Figure 4 A, B,). Interestingly, we found a decrease in the spontaneous activity of latent cells and a complete reversal of the firing frequency pattern previously observed in control animals (frequency pattern: type IV>III>II>I); see Figure 3A and C and Table S1. In intact sinoatrial node the pacemaker true cells have lower frequency than the latent cells. This is a protection mechanism of re-excitement and reentry arrhythmias.

**Figure 5:**
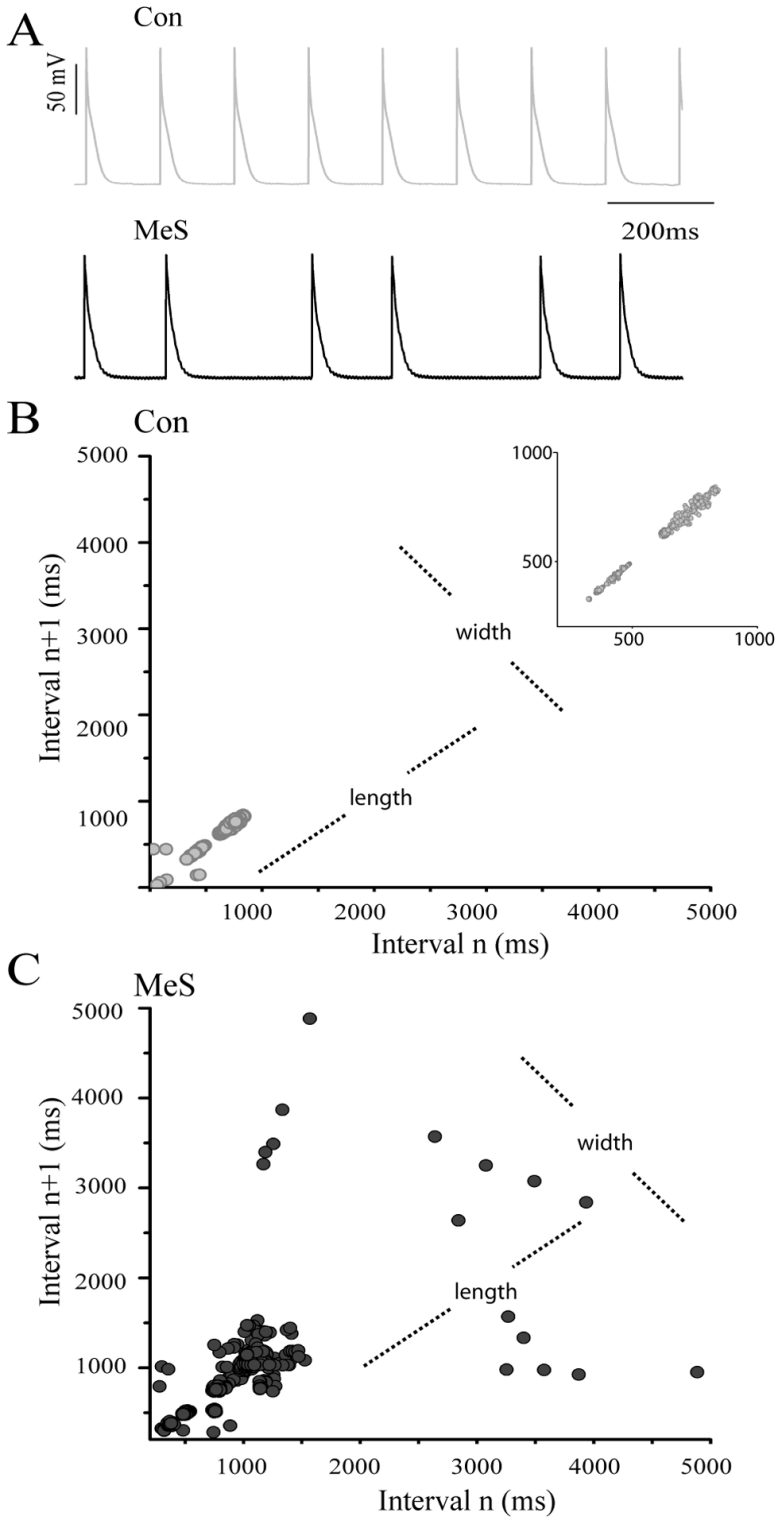
The MeS increase rate variability in the sinoatrial node. (**A**) Representative AP recorded on sinoatrial nodes from control and MeS animals, respectively. Note in the Poincaré plots a high variability between AP intervals recorded in rats with MeS (**C**) vs. control (**B**). The insert in B is the plot rescaling, “width” vs “length”. SD1/SD2 increased more than two times in MeS rats (control n = 11; MeS n = 13, p<0.05), indicating the possibility of suffering mortal arrhythmias.

In MeS rats, true nodal pacemaker cells have higher spontaneous activity than latent cells, which predispose nodal tissue to suffer arrhythmias (Figure 4A, 4C and 5). Accordingly, MeS increased the variability firing rate in nodal tissue, evidenced in the Poincaré plot (Control: SD1/SD2 = 0.25; MeS: SD1/SD2 = 0.55; Figure 5). In general, the frequency nodal tissue was reduced (Control: 2.04±0.21 Hz; MeS: 1.34±0.54 Hz, p>0.05). Also, MeS increased the slope of phase 4 in type II, III and IV cells (Figure 4A and 4C).

**Figure 6:**
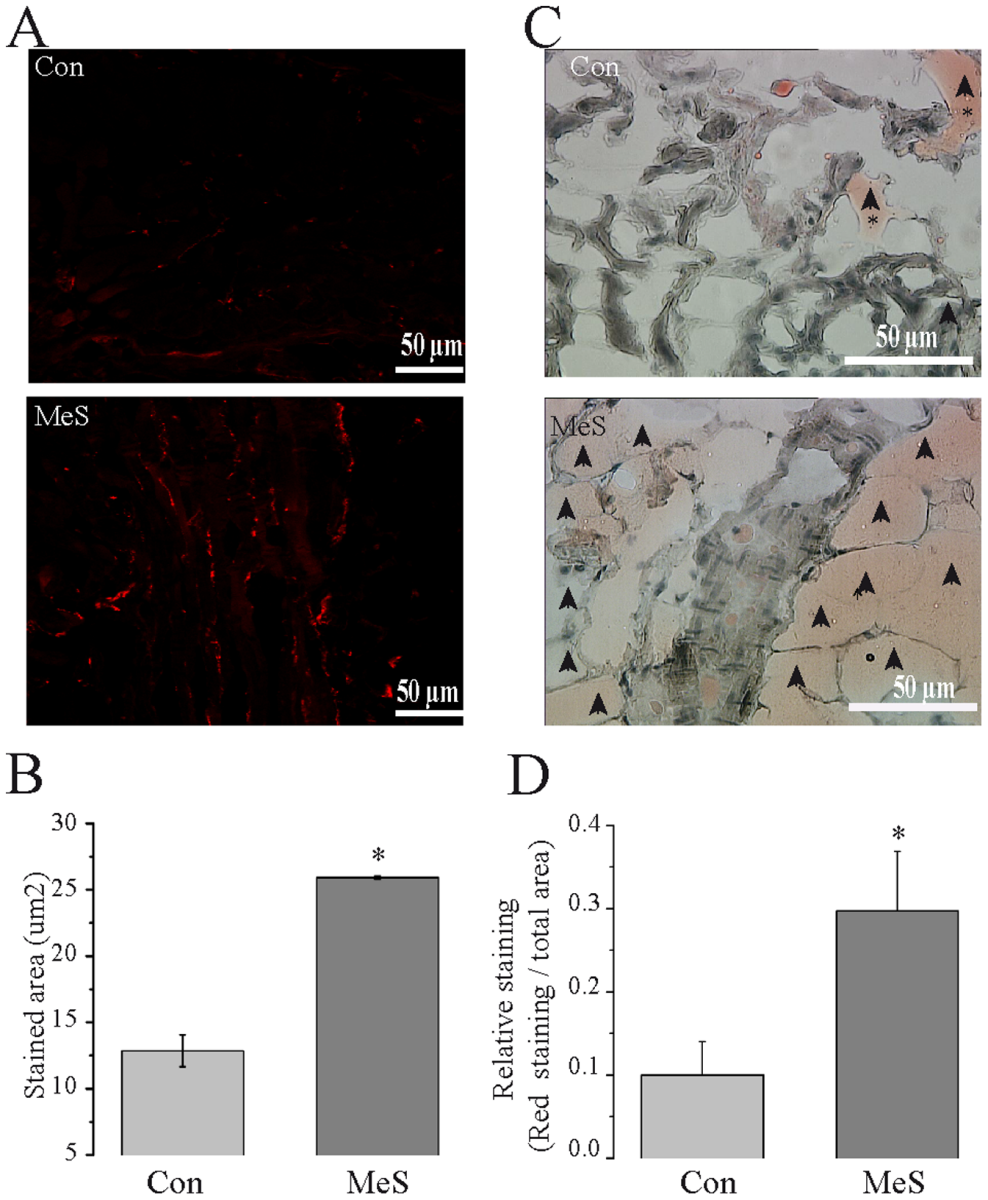
MeS increases adipose tissue and sympathetic innervation in sinoatrial node. (**A**) Immunofluorescence for tyrosine hydroxylase (TH) evidenced the sympathetic innervation of SA node. (**C**) In the same way, red oil staining showed adipose tissue surrounding nodal cardiomyocytes (arrows). (**B**) Quantification of stained areas revealed a major innervation and (**D**) a net increase of adipose tissue in SA nodes coming from rats with MeS (white arrows). Con: Control (n = 6); MES: MeS (n = 6).* p≤0.05 vs. control.

On the other hand, Figure 6 A and B show that immunoreactivity to tyrosine hydroxylase (TH), which is the first enzyme of catecholamine biosynthesis increased, compared to controls. In addition, sinoatrial nodes from MeS rats have three times more adipose tissue than control nodes. It is worth noting that this increase in fat is not only around the boundaries of nodal tissue but also in the center, surrounding the nodal cardiomyocytes (Figure 6C, D).

### MeS modified the voltage clock

**Figure 7:**
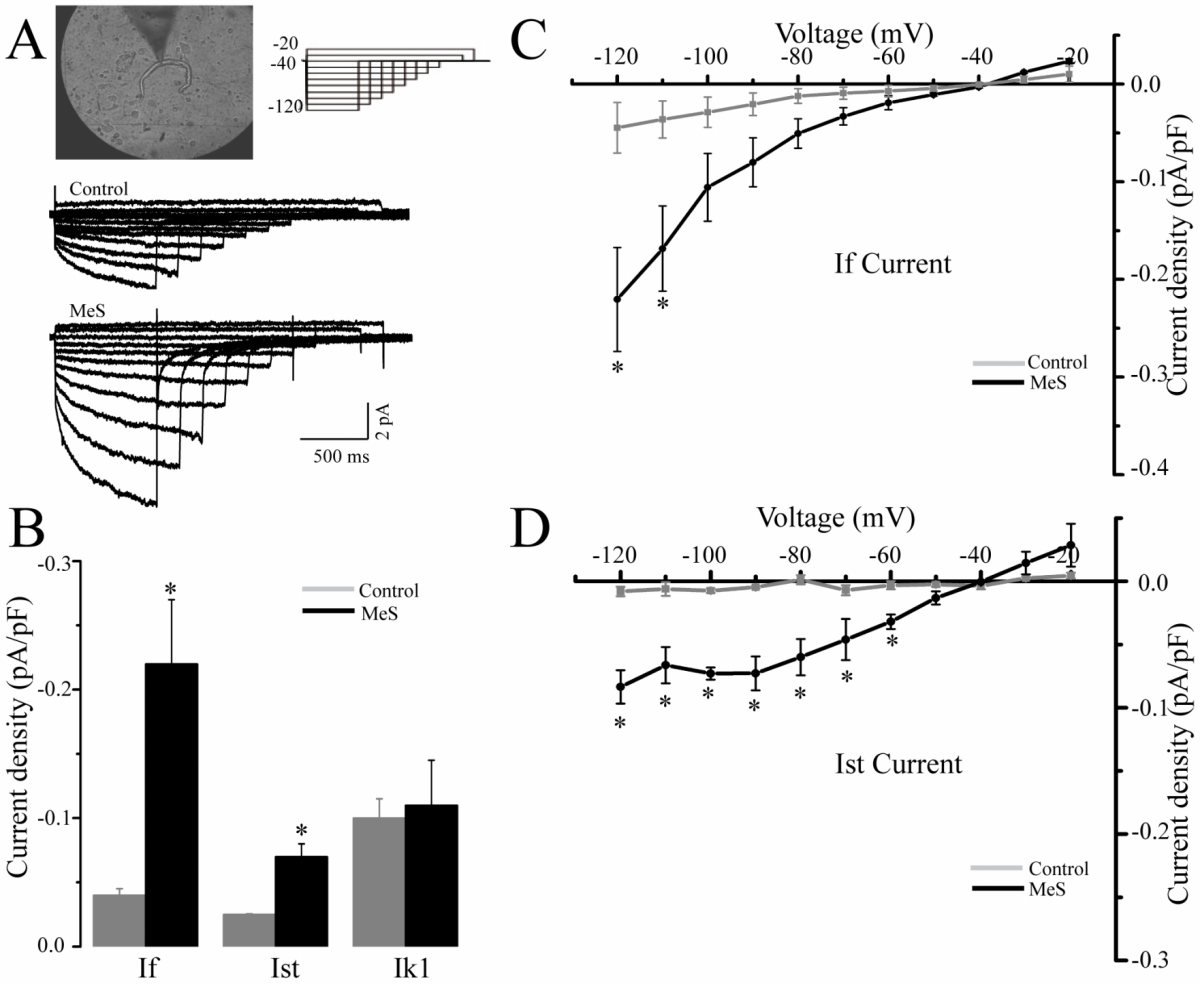
The pacemaker currents experiment remodeling on rats with MeS. (**A**) Patch clamp recorded cells were spindle shaped (upper left panel) with mean capacitances of 65±3 pF in control cells (n = 16) against 65±7 pF in the MeS cells (n = 16). Upper right panel shows the voltage protocol used to evocate I_f_ current. Inferior panel showed I_f_ current family control (con) and MeS, respectively (**B**). Bar graphs showing an increase I_f_ and I_st_, current density recorded at −120 mV in nodal cells from control (grey) and MeS (black) rats. Voltage vs. current graphs of I_f_ and I_st_ current **C** and **D** are respectively. * p<0.05 vs. control current evocated at the same voltage step.

The slow depolarizing 4 phase of the nodal AP in control rats depends of the I_f_ current, and two cationic currents, the I_st_ and the I_k1_. These three together are activated by hyperpolarizing potentials [24]. As shown in Figure 7, I_k1_ is the main hyperpolarizing current, followed closely by I_f_ (Figure 7A and 7B).

In rats with MeS, the main pacemaker current was I_f_, which was three-fold larger compared to controls (Figure 7A, B and C) at −120 mV. Furthermore, the I_st_ current also increased from 0.05 pA/pF to 0.1 pA/pF (Figure 7B and D) at the same voltage. The later correlates with the increase in the slope of phase 4 found in type II, III and IV AP.

## Discussion

This work demonstrates that MeS produced changes in nodal architecture and electrical activity, generating a dysfunction SA node, which could explain the onset of arrhythmias in rats.

Rat SA node has not been well characterized; almost all electrophysiological data in the literature are referred to rabbit SA node [22], [26]. The reports in rat were restricted to the tissue that surrounds the nodal artery, which was used as an anatomical reference. The electrophysiological characterization of this area showed several subtypes of AP [25], [26]. In this work, we found four different types of AP, based on several electrophysiological parameters, and in contrast to the previous reports, we also found true pacemaker cells and three different latent cell subtypes, distributed throughout the entire nodal surface (Figure S1).

To test the possible changes induced by MeS, we developed a model that mimics the etiology and signs of MeS in patients, by increasing sucrose intake in the drinking-water. Other MeS models have been studied [21], the most common being the mouse lacking leptin (ob^−^/ob^−^ mouse) [27]. These models develop hypertension that could *per se* modify all the cardiac functions, due to alterations in the morphology of the heart and vessels [28]. The present MeS model only develops mild hypertension and more than three other signs of MeS; obesity, impaired fasting glucose and high levels of insulin (Figure 1) [21].

The limitations of this model include that in the animal model we can select a single variable to be changed, in this case Wistar rats do not show a genetic predisposition to develop obesity and MeS and we induced it only increasing sucrose intake. In human subjects the scenario is quite different because of the complex genetic traits, a mixed excessive diet and differences in metabolism. For example, humans with MeS often show high concentrations of uric acid that rats do not develop (unpublished observations).

Other limitations to this work include that ECG signal morphology of the control rats is different to the human, because S and T waves are closer to the R-wave in rats.

We did not study the mechanism that altered the activity electrical; for example the managing of intracellular calcium “calcium clock” in the nodal cardiomyocytes. On the other hand, we could also consider possible changes in the extracellular matrix and in the action potential propagation on nodal tissue and pacemaker atrial cells. Finally other pathway to be studied is the intracellular signaling.

MeS produced an increase in R-R interval variability in the EGC (Figure 2), which is clinically manifested as arrhythmias. Moreover, the fact that R-R and P-P intervals have the same variability and no changes in the PQ interval were observed, indicate that in MeS rats sinus rhythm is never lost, and arrhythmias originate in the sinoatrial node (Figure S3). This is consistent with the Poincaré plot of the interpotential interval of spontaneous electrical activity (Figure 5), suggesting that in MeS rats augments the probability of arrhythmias like atrial fibrillation (AF) and sick sinus syndrome and ventricular fibrillation. We propose that these arrhythmias originate in the pacemaker.

We observed fat accumulation in the area node, as well as an increase in sympathetic innervation that could partly explain the remodeling of the tissue and the increment in phase 4 and the decrease APD by 30% and 60%. Accordingly, it has been observed that free fatty acids can modulate cardiac hyperpolarizing and potassium currents [29]. Similarly, Yanni et al, 2010 demonstrated that in old obese rats, the localization and morphology of true pacemaker AP shifts towards the inferior vena cava. It has been informed that patients with MeS have a decrease in sympathetic activity [3], [7]. This could be due to nerve growth factor (NGF) release by adipocytes; which could also explain the increase of innervation in the nodal tissue [30].

It is well known that the I_f_ current is positively modulated by free fatty acids and sympathetic innervation, thus predisposing cells to higher intracellular calcium levels, and therefore, increasing I_st_ current that are calcium dependent [10], [29]. Eventually the cardiomyocyte-fibroblast interactions are replaced by cardiomyocyte-adipocyte interactions, which decrease the electrotonic interaction between cardiomyocytes, thus uncoupling pacemaker cells [31].

In addition, in this MeS rat model, the pacemaker current is increased and the cells could be uncoupled by the adipocytes present around them that also change the conduction of electrical activity in the SA node. If we analyze the SA node as an oscillator commanded by the true pacemaker, this decoupling could be a risk factor for generating nodal arrhythmias. MeS rats develop four distinct oscillators, each one with different frequencies, originating variability in firing pattern frequency in the intact SA node (Figure 4 and 5).

We conclude that MeS modify the activity of the SA node by changing sympathetic innervation, remodeling the anatomy and the equilibrium between the different pacemaker currents; these effects predispose the sinoatrial node and heart to suffer arrhythmias.

## Supporting Information

Figure S1 **Sinoatrial node dissection.** (**A**) Sinoatrial node dissection was limited by the following structures: in the upper and lower side by the superior and inferior vena cava (SVC and IVC), on the left and right side by right atrium and the inter-atrial septum (IAS) (red dotted line). NA: nodal artery; NAb: nodal artery branches (yellow lines). (**B**) (B) Picture of sinoatrial node where are depicted the areas reported by other authors as the zone of true pacemaker cells in rabbit (gridded rectangle), mouse (squared oval) and rat (ellipse on nodal artery). Small box area = 1 mm^2^.
(TIF)

Figure S2 **Classification of sinoatrial node action potentials recorded in control rats.** Nodal action potentials were classified using three different parameters: amplitude, upstroke velocity and action potential duration (APD). The 3D graph shows the four types of the action potentials detected in sinoatrial node: True pacemaker action potential (black), type II (red), type III (blue) and type IV (green).
(TIF)

Figure S3 **SA node is the origin of arrhythmias in MeS rats.** (A) Poincaré plot using ECG PP interval. PP interval variability mimics the pattern observed R-R interval of the electrocardiogram. (B) Times series of ECG PQ interval, MeS does not induce changes in PQ interval of EGC. Control (grey) = 8 rats; Mes (black) = 7 rats.
(TIF)

Table S1 **Action potential parameters measured from the rat SA node.**
(DOC)
