# Supplementary figures and images for: Metabolic Syndrome Remodels Electrical Activity of the Sinoatrial Node and Produces Arrhythmias in Rats
Source: PLoS One. 2013 Nov 8;8(11):e76534. doi: 10.1371/journal.pone.0076534 (PMC3826723; doi:10.1371/journal.pone.0076534)

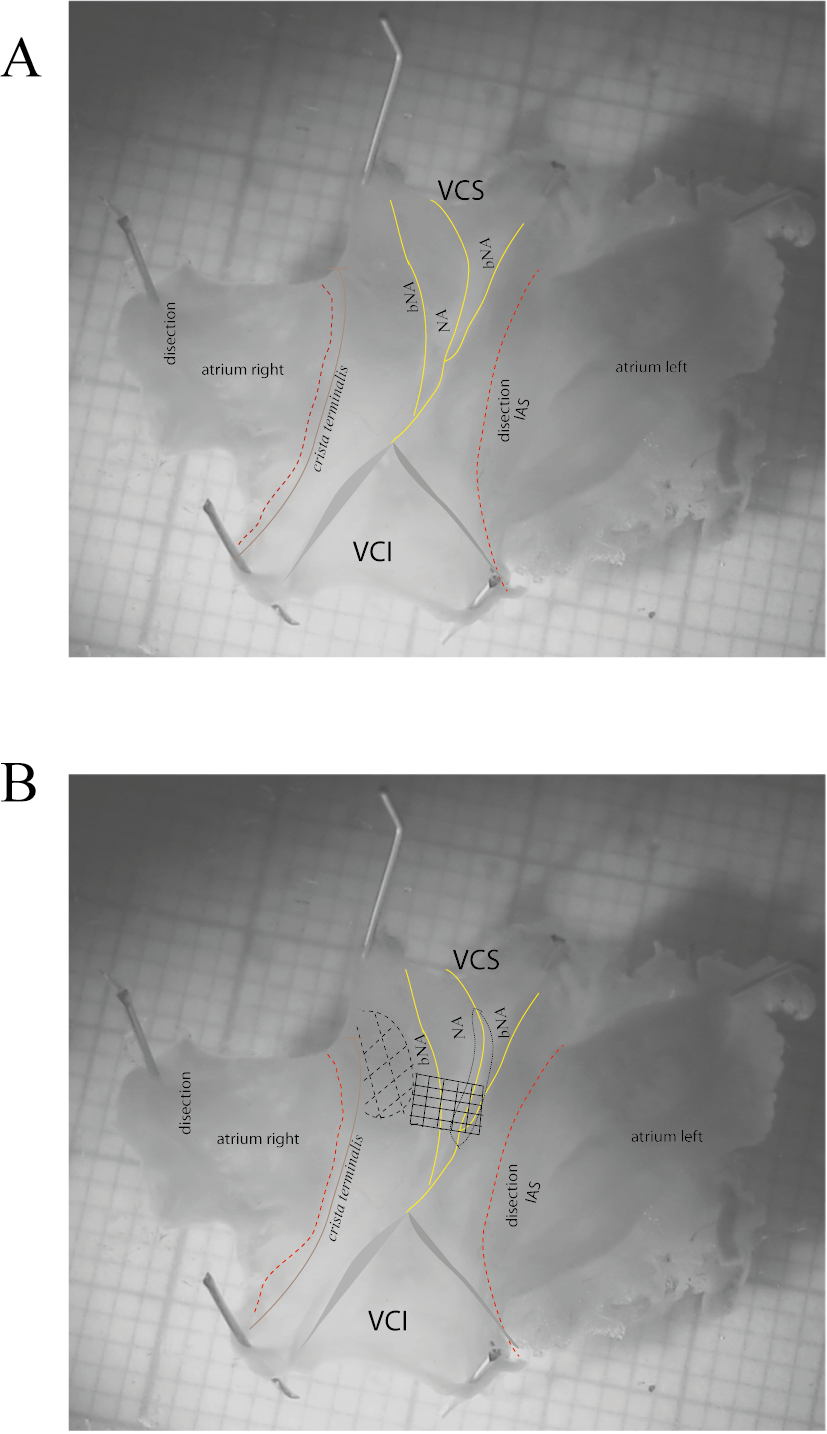

Supplement: Figure S1 — Sinoatrial node dissection. (A) Sinoatrial node dissection was limited by the following structures: in the upper and lower side by the superior and inferior vena cava (SVC and IVC), on the left and right side by right atrium and the inter-atrial septum (IAS) (red dotted line). NA: nodal artery; NAb: nodal artery branches (yellow lines). (B) (B) Picture of sinoatrial node where are depicted the areas reported by other authors as the zone of true pacemaker cells in rabbit (gridded rectangle), mouse (squared oval) and rat (ellipse on nodal artery). Small box area = 1 mm2. (TIF) [file pone.0076534.s001.tif]

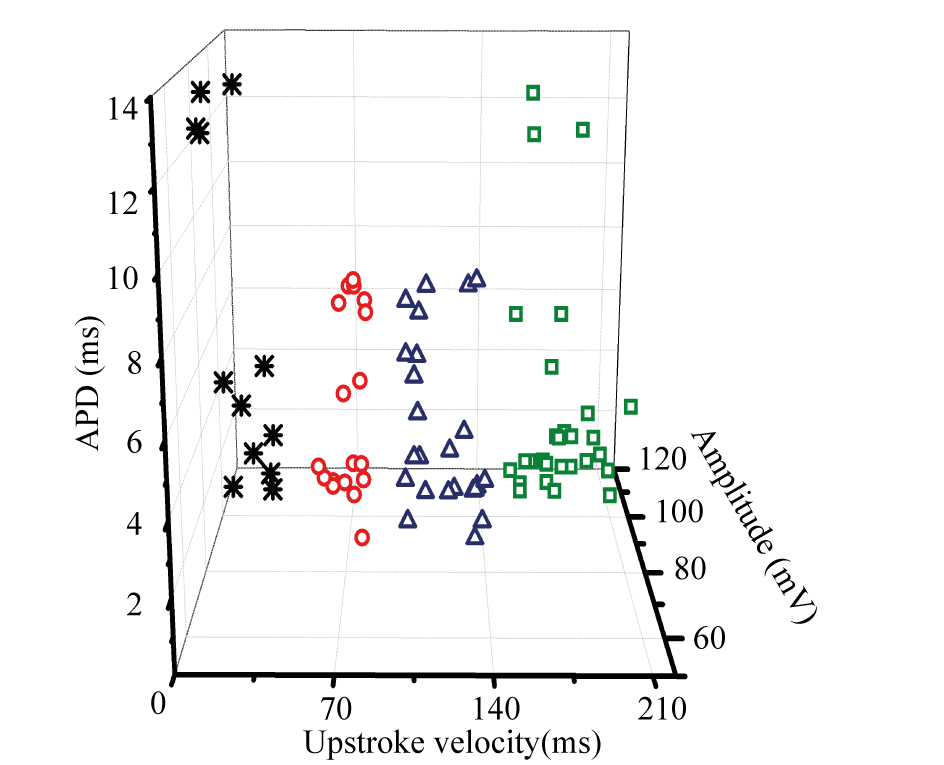

Supplement: Figure S2 — Classification of sinoatrial node action potentials recorded in control rats. Nodal action potentials were classified using three different parameters: amplitude, upstroke velocity and action potential duration (APD). The 3D graph shows the four types of the action potentials detected in sinoatrial node: True pacemaker action potential (black), type II (red), type III (blue) and type IV (green). (TIF) [file pone.0076534.s002.tif]

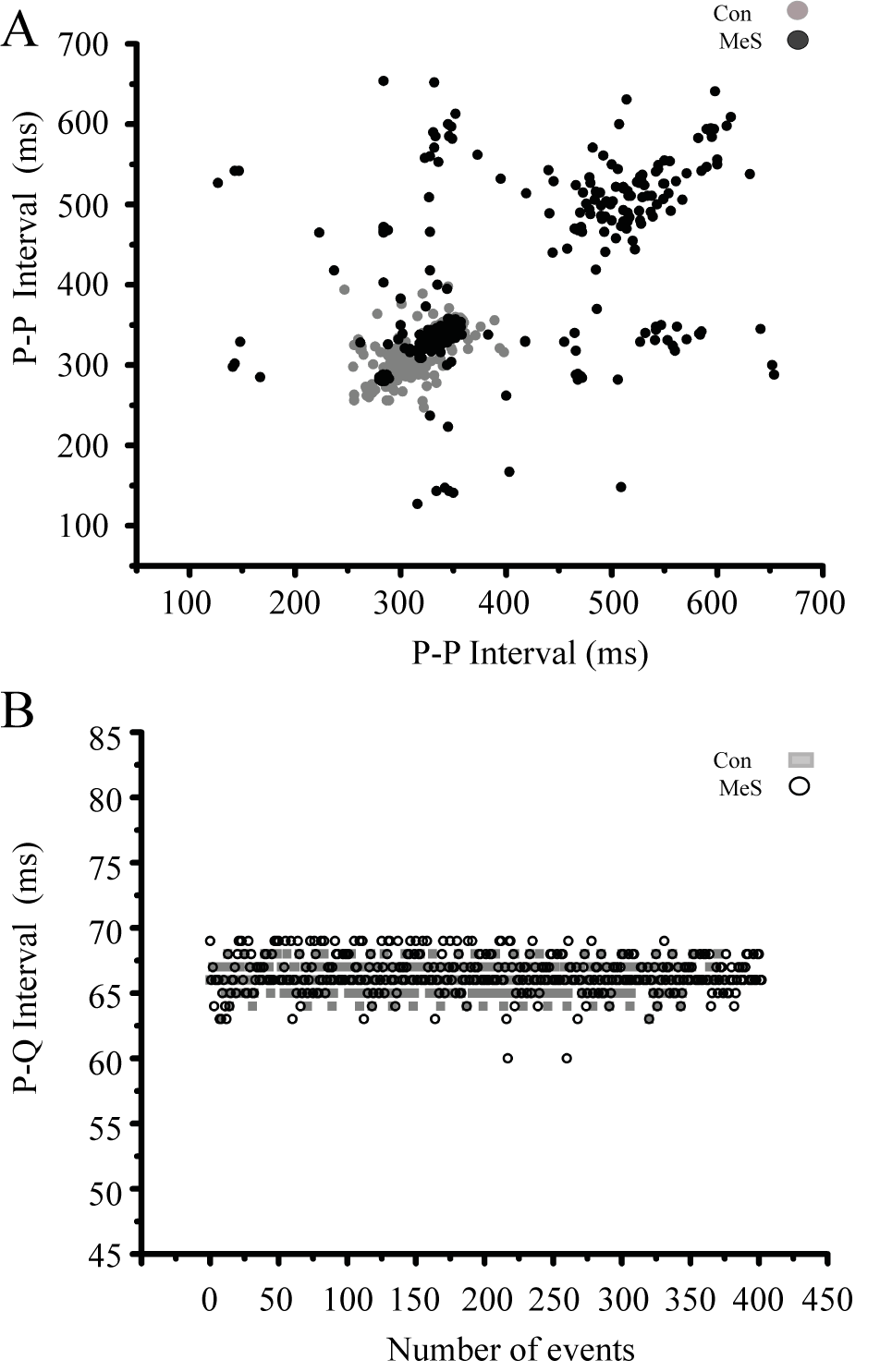

Supplement: Figure S3 — SA node is the origin of arrhythmias in MeS rats. (A) Poincaré plot using ECG PP interval. PP interval variability mimics the pattern observed R-R interval of the electrocardiogram. (B) Times series of ECG PQ interval, MeS does not induce changes in PQ interval of EGC. Control (grey) = 8 rats; Mes (black) = 7 rats. (TIF) [file pone.0076534.s003.tif]
