# Supplementary material for: Metabolic Syndrome Remodels Electrical Activity of the Sinoatrial Node and Produces Arrhythmias in Rats
Source: PLoS One. 2013 Nov 8;8(11):e76534. doi: 10.1371/journal.pone.0076534 (PMC3826723; doi:10.1371/journal.pone.0076534)
Supplement: Table S1 — Action potential parameters measured from the rat SA node. (DOC) [file pone.0076534.s004.doc]

Table S1. Action potential parameters measured from the rat SA node

|  |  | **True (nCon=17, nMes=11)** | **Latent II (nCon=14, nMes10)** | **Latent III (nCon=25, nMes=30)** | **Working IV (nCon=18, nMeS=15)** |
| --- | --- | --- | --- | --- | --- |
| **S (V/s)** | Con | 10 ± 2 | 0 € | 0 € | 0 € |
|  | Mes | 6 ± 4 | 0.4 ± 0.02* | 5 ± 0.1* | 0.7 ± 0.03* |
| **APD 30% (ms)** | Con | 18 ± 3 | 14 ± 2 € | 10 ± 2 £€ | 9 ± 2 €£≠ |
|  | Mes | 18 ± 3 | 10 ± 2* | 6 ± 1* | 6 ± 1 |
| **APD 60% (ms)** | Con | 47 ± 3 | 37 ± 3 € | 31 ± 3 £€ | 25 ± 3 €£≠ |
|  | Mes | 41 ± 6 | 34 ± 4 | 24 ± 3* | 28 ± 3 |
| **APD 90% (ms)** | Con | 104 ± 5 | 98 ± 4 | 82 ± 4£€ | 77 ± 3£€ |
|  | Mes | 83 ± 7 | 86 ± 8 | 82 ± 6 | 83 ± 6 |
| **TP (mV)** | Con | -58 ± 3 | -68 ±4€ | -73 ± 2€ | -77 ± 3 £€ |
|  | Mes | -53 ± 13 | -71 ± 3 | - 73 ± 2 | -75 ± 1 |
| **UV (V/s)** | Con | 31 ± 4 | 80 ± 4€ | 127 ± 3£€ | 179 ± 4 €£≠ |
|  | Mes | 16 ± 4* | 86 ± 5 | 123 ± 4 | 162 ± 2* |
| **R (Hz)** | Con | 2.2± 0.2 | 2.6 ± 0.1 | 2.7± 0.1€ | 2.8 ±0.05€ |
|  | Mes | 1.9± 0.3 | 1.4± 0.2* | 1.7 ±0.2* | 1.8± 0.2* |
| **A (mV)** | Con | 71± 18 | 82± 5 | 92± 7€£ | 104± 6€£≠ |
|  | Mes | 64± 5 | 77± 10 | 90± 7 | 102± 4 |

n, cell number; (Con, MS) control, metabolic syndrome; S, slope of diastolic depolarization; APD at 30,60,90% action potential duration at 30,60 or 90% repolarization; TP, threshold potential; UV, upstroke velocity R, spontaneous rate; A, amplitude. Statistically significant * control; € True; £ Latent II; ≠ Latent III pacemaker; p>0.05
